# Protein Composition of the Subretinal Fluid Suggests Selective Diffusion of Vitreous Proteins in Retinal Detachment

**DOI:** 10.1167/tvst.9.11.16

**Published:** 2020-10-14

**Authors:** Ebbe Toftgaard Poulsen, Xhevat Lumi, Anders K. Hansen, Jan J. Enghild, Goran Petrovski

**Affiliations:** 1 Department of Molecular Biology and Genetics, Aarhus University, Aarhus, Denmark; 2 Eye Hospital, University Medical Centre Ljubljana, Ljubljana, Slovenia; 3 Center for Eye Research, Department of Ophthalmology, Oslo University Hospital and University of Oslo, Oslo, Norway

**Keywords:** rhegmatogenous retinal detachment, subretinal fluid, proteomics, vitreous humor

## Abstract

**Purpose:**

To study the proteome of the subretinal fluid (SRF) from rhegmatogenous retinal detachment (RRD) in search for novel markers for improved diagnosis and prognosis of RRD.

**Methods:**

Human undiluted SRF obtained during vitrectomy for primary RRD using a 41-gauge needle (*n* = 24) was analyzed and compared to vitreous humor from 2-day postmortem eyes (*n* = 20). Sample preparation underwent nanoflow liquid chromatography–tandem mass spectrometry. Label-free quantification (LFQ) using MaxQuant was used to determine differentially expressed proteins between SRF and vitreous humor. The intensity-based absolute quantification (iBAQ) was used to rank proteins according to their molar fractions within groups. Identification of proteins beyond the quantitative level was performed using the Mascot search engine.

**Results:**

The protein concentration of the control vitreous humor was lower and more consistent (1.2 ± 0.4 mg) than that of the SRF (17.9 ± 22 mg). The iBAQ analysis showed high resemblance between SRF and vitreous humor, except for crystallins solely identified in vitreous humor. The LFQ analysis found 38 protein misregulations between SRF and vitreous humor of which the blood coagulation pathway was found to be enriched using the PANTHER Classification System. Combined, the iBAQ, LFQ, and Mascot analysis found an overlap only in chitinase-3-like protein 1 and galectin-3-binding protein unique to the SRF.

**Conclusions:**

The proteome of the SRF was highly represented by proteins involved in proteolysis. Such proteins can possibly serve as targets in modulating the effects of SRF in RD.

**Translational Relevance:**

To identify potential novel biomarkers for therapeutic targeting in RD.

## Introduction

Rhegmatogenous retinal detachment (RRD) is the most common vision-threatening retinal condition requiring urgent care. With its relatively high incidence of 1:10,000 to 15,000 per year, RRD affects mostly the elderly population as well as those in their mid/late working age.^1^^,^^2^ Delayed or improper surgical treatment of patients in the acute phase of the disease can cause severe to permanent visual impairment.

RRD can be described by two main cellular mechanisms: inflammation and cell death. The inflammation is sterile without any presence of infection. Fresh detachment is hallmarked by the presence of hyperreflective points on optical coherence tomography at the detached neuroretina/retinal pigment epithelium (RPE) border, which at later stages likely contain aggregates of macrophages or activated microglia (CD68^+^ C34^−^) at this cellular interface.^3^ Vitreoretinal traction plays a role in RRD by allowing accumulation of liquefied vitreous under the retina, leading to its separation from the RPE. According to this, there are critical preconditions or mechanisms that lead to this type of retinal detachment (RD), such as presence of liquefied vitreous, tractional forces, and a break through which fluid gains access to the subretinal space.^4^^,^^5^ Cell death in RRD induced by consequent ischemia, for the most part, takes place in the photoreceptors,^6^ typically of the type I or apoptotic form, but it can also be in the form of programmed necrosis, cytokine-dependent, and autophagy-associated cell death.^7^

The pathomechanisms of RRD can be divided into actions occurring during the acute phase of the detachment and actions during the chronic phase, which is hallmarked by proliferative vitreoretinopathy (PVR). The earliest structural effects of RD can be seen on the outer segments (OSs) of the photoreceptors and RPE cells. Between these two structures, there are no actual cellular junctions in the mature eye, but they are adherent through the numerous microvilli present on the apical surface of the RPE. Upon RD, these connections become damaged, and a space between the two cellular layers is formed. The subretinal space is usually free of cellular content, but during RD, cells such as neutrophils, monocytes, and macrophages can migrate into the damaged area together with migrating RPE cells.^3^ The damaged OSs of the photoreceptors also flow into the subretinal space, where they become phagocytosed by RPE cells or other phagocytes present in the vicinity. Within 24 hours from RD, microglia-like cells have been shown to display signs of proliferation.^8^ Although neural stem/progenitor cells have been shown to be activated during retinal injury, only the population with Müller glia characteristics could undergo targeted migration into the vitreous.^9^

The subretinal fluid (SRF) found between the neuroretina and the underlying RPE layer during RD can be studied to determine its composition. It has been shown in limited cytokine/proteomics analysis studies that the SRF contains cytokines and other factors that could have been secreted as a cause or a consequence of the RD or as part of a sterile form of inflammation accompanying the process.^10^

In this study, we have analyzed the proteome of the SRF to find new molecular markers, which can help improve the diagnosis and prognosis of RD.

## Methods and Materials

### Sample Collection

Human undiluted SRF was obtained immediately before vitrectomy for primary RRD using a 41-gauge needle. The time interval from the first sign of RRD to vitrectomy ranged between 6 days and more than 6 months (Supplementary Table S1). Severity of the detachment was PVR grade C for all patients at the time of surgery. PVR stage was graded according to the updated classification of Retina Society Terminology Committee (1991).^11^ All 24 samples (6 females and 18 males, average age 68 years) were collected in sterile polypropylene tubes and stored at −80°C until analysis. Sample volumes ranged between 300 and 700 µL. Vitreous humor samples were obtained from 2-day postmortem eyes (*n* = 20, 11 females and 9 males, average age 83 years). The postmortem samples were obtained from 20 different donors with no history of ocular pathology (Supplementary Table 1). Samples were handled in accordance with the tenets of the Declaration of Helsinki, and informed consent was obtained from patients undergoing surgery. The local institutional review board approved the research.

### Sample Preparation for Liquid Chromatography–Tandem Mass Spectrometry

Human SRF and human vitreous samples were processed using filter-aided sample preparation (FASP). Protein concentrations were determined using the A280 option of the NanoDrop 2000c system (Thermo Fisher Scientific, Waltham, MA, USA) and the Bradford assay (Bio-Rad, Hercules, CA, USA). The pH of samples was roughly estimated using pH strips (Merck Millipore, Burlington, MA, USA). For each FASP digest, 20 µg of each sample was applied to a 10-kMw cutoff filter (Millipore) and centrifuged at 14,000 × *g* at 22°C until almost dry. The concentrated sample was resuspended in 200 µL 6 M urea and 100 mM ammonium bicarbonate (pH 8.0), followed by centrifugation at 14,000 × g for 30 minutes. The sample was then reduced by adding 20 µL 500 mM dithiothreitol (DTT) and 100 mM ammonium bicarbonate (pH 8.0) to the filter, incubated for 5 minutes, and spun another 14,000 × g for 30 minutes. Thiol-groups were alkylated by adding 20 µL 500 mM iodoacetamide in 100 mM ammonium bicarbonate (pH 8.0), vortexed, and incubated for 5 minutes in the dark at 22°C. Excess iodoacetamide was removed by centrifugation at 14,000 × g, followed by a washing step consisting of 200 µL 100 mM ammonium bicarbonate (pH 8.0) before being centrifuged at 14,000 × g for 30 minutes. Last, the washed and concentrated sample was added to 100 µL 100 mM ammonium bicarbonate (pH 8.0) containing 250 ng MS-grade trypsin (Sigma-Aldrich St. Louis, MO, USA), vortexed, and placed at 37°C for 16 hours. The following day, the filtrate was collected in a new tube and acidified by adding 10 µL 5% formic acid. Samples were desalted using homemade RP micro-columns plugged with Octadecyl C18 Solid Phase Extraction disks (3M, Maplewood, MN, USA) and dissolved in 0.1% formic acid before liquid chromatography–tandem mass spectrometry (LC-MS/MS) analysis.

### LC-MS/MS Analysis

The samples for mass spectrometry were analyzed by nanoflow LC-MS/MS using an Eksigent nanoLC 415 system (SCIEX, Framingham, MA, USA) connected to a TripleTOF 6600 mass spectrometer (SCIEX, Framingham, MA, USA) equipped with a NanoSpray III source (SCIEX, Framingham, MA, USA). The nanoLC was fitted with 0.1 × 20-mm 3-µm C18 trap column and 0.075 × 150-mm 3-µm C18 analytical columns, both pulled and packed in-house using ReproSil-Pur C18-AQ 3-µm resin (Dr. Marisch GmbH, Ammerbuch-Entringen, Germany). The chromatographic separation of peptides was carried out at a flow rate of 250 nL/min using a 50-minute linear gradient from 5% to 35% B solvent (0.1% formic acid and 90% acetonitrile) followed by 10 minutes at 95% B solvent. Data were acquired using an ion spray voltage of 2.6 kV, a curtain gas of 35, and an interface heater temperature of 150°C. For data-dependent acquisition (DDA), survey scans were acquired in 250 ms over a mass range of 300 to 1800 m/z. Up to 50 product ion scans were collected, using dynamic exclusion of 12 seconds, if exceeding a threshold of 100 counts per second and with a +2 to +5 charge state. A sweep collision energy setting of 35 ± 15 eV was applied to all precursor ions for collision-induced dissociation. The cycle time of the DDA method was 1.55 seconds. resulting in minimum of eight data points across the chromatographic peak, thereby allowing quantification using the extracted ion chromatography.

### MaxQuant iBAQ and LFQ Quantification

MaxQuant (version 1.6.5.0; www.maxquant.org) was used to generate mascot generic files, and the Andromeda algorithm interrogated the SwissProt human database (2019_4; 20,422 sequences) using the following parameters: carbamidomethyl and *N*-terminal acetylation as fixed modifications and oxidation of methionine and proline as variable modifications. Trypsin was selected as the digestion enzyme allowing two missed cleavages. The false discovery rate (FDR) of peptides and proteins was set to 1%, and the mass tolerances of first and main precursor searches were set at 0.07 and 0.006 Da. The peptide mass tolerance was set at 20 ppm. The MaxQuant software was used to calculate the label-free quantification (LFQ) and intensity-based absolute quantification (iBAQ) intensities for each protein based on a minimum of two peptides, including both unique and razor peptides. Match between runs was allowed among each group. LFQ and iBAQ intensities were processed and statistically compared using the Perseus software (version 1.6.5.0; www.maxquant.org). Values that were based on reverse sequence and only identified by site were removed. Only proteins quantified in a minimum of 75% of the replicates were included in the further comparison between groups. The molar percentage was calculated by dividing the iBAQ intensity of one protein by the total iBAQ intensity of the given sample. The proteins were ranked based on their average molar percentage in each group. For statistical analysis, the LFQ intensity values were logarithmized (Log2) and subjected to a Student's *t*-test, followed by multiple hypothesis correction using the Benjamini-Hochberg method and an FDR of 0.01. Protein regulation with an adjusted *P*-value (*q*-value) below 0.01 was regarded as regulated between groups. The SRF group was further divided into three subgroups consisting of (1) 1 to 7 days post-RD, (2) 8 to 30 days post-RD and, (3) >30 days post-RD. The SRF subgroups were compared with each other and to the vitreous humor group, using same statistics as above, but relying on proteins to be quantified in a minimum of five samples within each group.

### Mascot Protein Identification

The total protein identification in each group was obtained using the Mascot search engine v.2.5.1 interrogating the SwissProt human database (2019_7; 20,432 sequences). Precursor and production tolerance were set to 15 ppm and 0.2 Da, respectively. Trypsin was specified as the enzyme allowing one missed cleavage. Carbamidomethyl was selected as fixed modification and oxidation of methionine and proline as variable modifications. Search results was adjusted to a 1% FDR at the protein level and imported to MS Data Mine v.1.3^12^ for comparison.

### Ontology and Pathway Analyses

The PANTHER Classification System v.15.0^13^ was used to categorize proteins into molecular function using *Homo* *sapiens* as a reference organism and UniProt accession numbers as entries. Furthermore, a statistical overrepresentation test, using the Panther Pathways annotation, was performed for LFQ regulated proteins of the SRF group. Test parameters were *H.* *sapiens* as a reference list, while Fisher exact test was selected as the test of choice, and the Benjamini-Hochberg calculated the FDR of *P* < 0.05 to correct for multiple hypothesis testing.

## Results

### SRF Does Not Reflect the Vitreous in Protein Concentration

**Figure 1.**
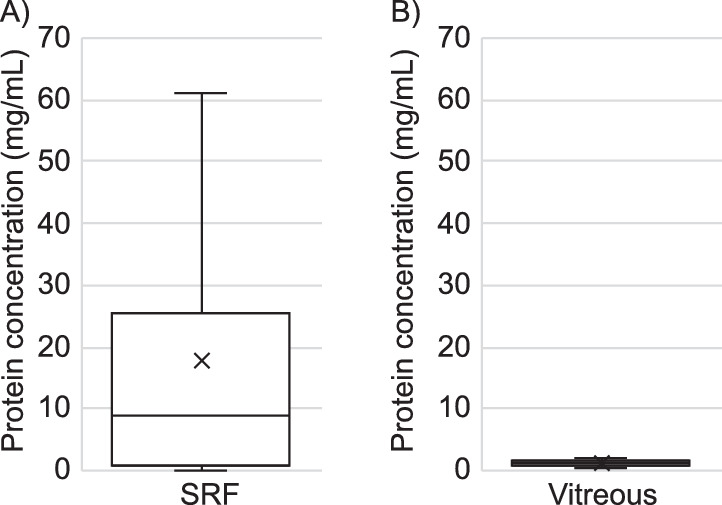
The protein concentration in SRF is high compared to vitreous humor. Box-and-whiskers plot showing the distribution of protein concentration of (A) SRF and (B) vitreous. (A) The SRF samples had a mean ± SD of 17.9 ± 22 mg/mL and displayed a maximum and minimum of 61.0 and 0.02 mg/mL, respectively. (B) The 20 vitreous samples had a mean ± SD protein concentration of 1.2 ± 0.4 mg/mL and a maximum and minimum of 2.0 and 0.6 mg/mL, respectively.

A proteomic comparison of 24 human biological replicates of SRF from RRD with 20 two-day postmortem human biological replicates of vitreous humor was performed. While vitreous humor showed a more consistent protein concentration crosswise (1.2 ± 0.4 mg/mL), the protein concentration of the SRF samples (17.9 ± 22 mg/mL) deviated to a much larger extent (Fig. 1). The inconsistency in protein concentrations between SRF and vitreous humor suggests that accumulation of fluid behind the neuroretinal layer relies on more than passive diffusion of vitreous into the subretinal space.

### Label-Free Quantification of the Most Abundant Proteins

**Figure 2.**
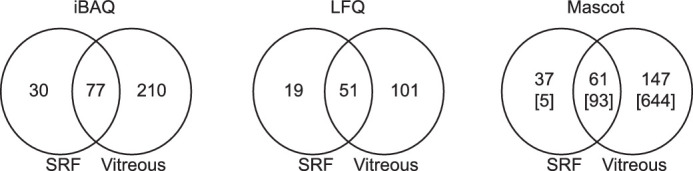
Venn diagram of iBAQ, LFQ, and Mascot analyses. For iBAQ and LFQ, data rely on quantification in a minimum of 75% of the samples in each group. For the Mascot qualitative analysis, the proteins were identified in a minimum of 50% of the samples in each group. The numbers in squared brackets define proteins identified in the minimum half of the SRF group and any of the samples in the vitreous control group.

To analyze the resemblance between SRF and vitreous humor, two label-free proteomics approaches were applied. The first approach, iBAQ, compared the molar percentage within each group, allowing one to compare the major protein constituents of SRF and vitreous, respectively (Table 1). In the vitreous, 287 proteins were quantified, whereas 107 proteins were quantified in SRF. A total of 77 proteins were shared between vitreous and SRF (Fig. 2). Of the 50 most abundant proteins in SRF, 18 also appeared as top 50 proteins in the vitreous; 28 proteins were quantified in both SRF and vitreous but only found as top 50 proteins in SRF; the remaining 4 proteins of the 50 most abundant ones in SRF, Ig lambda-6 chain C region (UniProt: P0DOY3), Ig heavy chain V-III region CAM (UniProt: P0DP03), Ig kappa chain V-III region POM (UniProt: A0A0C4DH55), and keratin type II cytoskeletal 1 (UniProt: 0A0C4DH55), were only quantified in SRF but not in the vitreous.

**Table 1.** The 50 Most Abundant Proteins in Subretinal Fluid and Vitreous Humor

Vitreous			SRF		
Accession	Name	Mol %	Accession	Name	Mol %
**P02768**	Serum albumin	37.0 ± 14.8	**P02768**	Serum albumin	58.5 ± 7.5
A0A140G945*	Alpha-crystallin A chain	4.5 ± 6.5	**P02787**	Serotransferrin	5.3 ± 1.5
**P01009**	Alpha-1-antitrypsin	4.2 ± 2.3	**P01834**	Ig kappa chain C region	4.6 ± 3.1
**P02787**	Serotransferrin	3.9 ± 1.6	**P01857**	Ig gamma-1 chain C region	3.4 ± 1.0
**P01857**	Ig gamma-1 chain C region	2.3 ± 1.5	**P02647**	Apolipoprotein A-I	3.0 ± 1.0
**P41222**	Prostaglandin-H2 D-isomerase	2.1 ± 1.3	**P01009**	Alpha-1-antitrypsin	2.9 ± 0.9
P02511*	Alpha-crystallin B chain	2.1 ± 3.6	**P10909**	Clusterin	2.1 ± 0.9
P06733*	Alpha-enolase	1.8 ± 1.2	**P41222**	Prostaglandin-H2 D-isomerase	1.6 ± 0.8
P43320*	Beta-crystallin B2	1.7 ± 3.1	**P02652**	Apolipoprotein A-II	1.5 ± 0.6
**P02763**	Alpha-1-acid glycoprotein 1	1.5 ± 1.1	**P36955**	Pigment epithelium-derived factor	1.4 ± 0.7
P29762*	Cellular retinoic acid-binding protein 1	1.5 ± 0.9	**P10745**	Retinol-binding protein 3	1.3 ± 1.1
P08670*	Vimentin	1.4 ± 1.4	**P02790**	Hemopexin	1.0 ± 0.4
**P10909**	Clusterin	1.4 ± 0.7	**P01011**	Alpha-1-antichymotrypsin	1.0 ± 0.5
P04406*	Glyceraldehyde-3-phosphate dehydrogenase	1.3 ± 0.9	**P01034**	Cystatin-C	1.0 ± 0.6
**P36955**	Pigment epithelium-derived factor	1.3 ± 0.8	**P02763**	Alpha-1-acid glycoprotein 1	1.0 ± 0.4
**P02647**	Apolipoprotein A-I	1.2 ± 0.9	P02765	Alpha-2-HS-glycoprotein	0.8 ± 0.5
**P01834**	Ig kappa chain C region	1.0 ± 0.9	**P68871**	Hemoglobin subunit beta	0.8 ± 2.7
P12277*	Creatine kinase B-type	1.0 ± 0.6	P01859	Ig gamma-2 chain C region	0.5 ± 0.4
P63261	Actin, cytoplasmic 2	0.9 ± 0.6	P01876	Ig alpha-1 chain C region	0.5 ± 0.5
P00918*	Carbonic anhydrase 2	0.9 ± 0.5	P07339	Cathepsin D	0.4 ± 0.3
**P68871**	Hemoglobin subunit beta	0.9 ± 0.6	P02766	Transthyretin	0.4 ± 0.2
**P01034**	Cystatin-C	0.9 ± 0.5	P01860	Ig gamma-3 chain C region	0.4 ± 0.2
**P01011**	Alpha-1-antichymotrypsin	0.9 ± 0.6	B9A064	Immunoglobulin lambda-like polypeptide 5	0.3 ± 0.2
P69905*	Hemoglobin subunit alpha	0.7 ± 0.6	P06727	Apolipoprotein A-IV	0.3 ± 0.1
P30086*	Phosphatidylethanolamine-binding protein 1	0.7 ± 0.3	P02774	Vitamin D-binding protein	0.3 ± 0.1
P0DOY2*	Ig lambda-6 chain C region	0.7 ± 0.6	P05090	Apolipoprotein D	0.3 ± 0.3
Q93077*	Histone H2A type 1-C	0.7 ± 1.4	**P02649**	Apolipoprotein E	0.3 ± 0.1
**P02790**	Hemopexin	0.6 ± 0.5	P08100	Rhodopsin	0.3 ± 0.6
P14618*	Pyruvate kinase PKM	0.6 ± 0.4	**P00738**	Haptoglobin	0.3 ± 0.4
P62807*	Histone H2B type 1-C/E/F/G/I	0.6 ± 1.2	P00450	Ceruloplasmin	0.2 ± 0.1
P60174*	Triosephosphate isomerase	0.5 ± 0.3	P04217	Alpha-1B-glycoprotein	0.2 ± 0.1
**P02652**	Apolipoprotein A-II	0.5 ± 0.4	P22352	Glutathione peroxidase 3	0.2 ± 0.2
P09104*	Gamma-enolase	0.5 ± 0.3	P61769	Beta-2-microglobulin	0.2 ± 0.2
P62805*	Histone H4	0.5 ± 1.1	P19652	Alpha-1-acid glycoprotein 2	0.2 ± 0.1
P84243*	Histone H3.3	0.5 ± 1.0	P0DOY3*	Ig lambda-6 chain C region	0.2 ± 0.1
P22914*	Beta-crystallin S	0.5 ± 0.9	P11488	Guanine nucleotide-binding protein G(t) subunit alpha-1	0.2 ± 0.2
**P00738**	Haptoglobin	0.5 ± 0.7	P05155	Plasma protease C1 inhibitor	0.2 ± 0.1
P10523	S-arrestin	0.5 ± 0.3	P01024	Complement C3	0.1 ± 0.1
**P10745**	Retinol-binding protein 3	0.4 ± 0.2	Q9UBP4	Dickkopf-related protein 3	0.1 ± 0.1
P62328*	Thymosin beta-4	0.4 ± 0.2	P01619	Ig kappa chain V-III region B6	0.1 ± 0.1
P68363*	Tubulin alpha-1B chain	0.4 ± 0.4	P0DP03*	Ig heavy chain V-III region CAM	0.1 ± 0.3
P14136*	Glial fibrillary acidic protein	0.4 ± 0.4	P10451	Osteopontin	0.1 ± 0.1
P00338*	L-lactate dehydrogenase A chain	0.4 ± 0.3	P0C0L4	Complement C4-A	0.1 ± 0.1
P68371*	Tubulin beta-4B chain	0.4 ± 0.4	P06396	Gelsolin	0.1 ± 0.0
P12271*	Retinaldehyde-binding protein 1	0.3 ± 0.3	P04264*	Keratin, type II cytoskeletal 1	0.1 ± 0.3
P14174*	Macrophage migration inhibitory factor	0.3 ± 0.2	P01023	Alpha-2-macroglobulin	0.1 ± 0.1
P00558*	Phosphoglycerate kinase 1	0.3 ± 0.2	P01008	Antithrombin-III	0.1 ± 0.1
P62937*	Peptidyl-prolyl cis-trans isomerase A	0.3 ± 0.2	Q9UBM4	Opticin	0.1 ± 0.1
P20962*	Parathymosin	0.3 ± 0.3	A0A0C4DH55*	Ig kappa chain V-III region POM	0.1 ± 0.1
**P02649**	Apolipoprotein E	0.3 ± 0.1	P02749	Beta-2-glycoprotein 1	0.1 ± 0.0

Bold defines top 50 quantified proteins shared between SRF and vitreous groups.

Nonbold without asterisk are shared between groups but only as top 50 in either SRF or vitreous.

Asterisk defines proteins only quantified in either the SRF or vitreous groups.

Extended information on search results is found in SI Table 3.

Data listed in Table 1 rely on protein identification in a minimum of 75% of the samples, which means that the four top 50 proteins solely quantified in SRF of the iBAQ analysis may have been identified in some of the vitreous samples. We, therefore, used a more stringent criterion looking at proteins quantified in at least 75% of the SRF samples but not in any of the vitreous samples. This comparison resulted in 13 proteins uniquely quantified in SRF using the iBAQ analysis (Table 2).

**Table 2.** Proteins Quantified Solely in SRF in the iBAQ, LFQ, and Mascot Analyses

Accession	Name	iBAQ	LFQ	Mascot
Q96JP9	Cadherin-related family member 1	X		X
P36222	Chitinase-3-like protein 1	X	X	X
Q15846	Clusterin-like protein 1	X		
P00748	Coagulation factor XII	X		
P02747	Complement C1q subcomponent subunit C	X		X
Q08380	Galectin-3-binding protein	X	X	X
P05546	Heparin cofactor 2	X		
A0A0C4DH55	Ig kappa chain V-III region POM	X		
P0DOY3	Ig lambda-6 chain C region	X	X	
P08779	Keratin, type I cytoskeletal 16	X	X	
P02538	Keratin, type II cytoskeletal 6A	X		
Q6EMK4	Vasorin	X		X
P07225	Vitamin K-dependent protein S	X		

The second quantitative approach used to analyze the proteomic data relied on the LFQ intensities calculated by MaxQuant. The LFQ analysis identified significant protein misregulations between SRF and vitreous. In total, 152 and 70 proteins were quantified in vitreous and SRF, respectively, of which 51 were in common (Fig. 2).

The LFQ analysis resulted in 38 protein regulations, of which only 3 were downregulated and the remaining 35 upregulated (Table 3). The LFQ comparison also relies on quantification in a minimum of 75% of the samples and thus only provides information on misregulation if proteins in both groups fulfilled this requirement. The SRF group was furthermore grouped into three subgroups, depending on the time from RD to surgery (group 1: 1–7 days post-RD; group 2: 8–30 days post-RD; group 3: >30 days post-RD), then compared across and to the vitreous humor group. No significant regulated proteins were found between the SRF subgroups. However, comparison of the SRF subgroups to the vitreous samples indicated progressive misregulation of proteins over time (Table 3). Similar to the iBAQ analysis, a more stringent criterion was also applied to the SRF analysis, looking at proteins only quantified in the SRF group fulfilling the 75% requirement but not in any of the vitreous samples. This resulted in four proteins, including chitinase-3-like protein 1 (UniProt: P36222), galectin-3-binding protein (UniProt: Q08380), immunoglobulin lambda constant 3 (UniProt: P0DOY3), and keratin 16 (UniProt: P08779) only quantified in SRF. The four proteins overlapped with the proteins unique for SRF in the iBAQ analysis (Table 2). While caution should be taken with keratins as they often appear as contamination during sample preparation, the two proteins, chitinase-3-like protein 1 and galectin-3-binding protein, may be important for the disease progression in RRD.

**Table 3.** LFQ Significant Regulated Proteins between SRF and Vitreous Groups

Accession	Name	*P*-Value	*q*-Value	Ratio (SRF/V)	1–7 Days Post-RD	8–30 Days Post-RD	>30 Days Post-RD	Regulation
P63261	Actin, cytoplasmic 2	2.5E-24	1.3E-22	0.1		X	X	↓
P19652	Alpha-1-acid glycoprotein 2	1.6E-04	2.6E-04	1.9		X		↑
P01011	Alpha-1-antichymotrypsin	6.9E-03	9.2E-03	1.6			X	↑
P04217	Alpha-1B-glycoprotein	2.0E-08	8.4E-08	3.0		X	X	↑
P02765	Alpha-2-HS-glycoprotein	1.0E-20	2.6E-19	8.8	X	X	X	↑
P01023	Alpha-2-macroglobulin	4.4E-04	6.6E-04	2.3	X			↑
P01019	Angiotensinogen	4.4E-12	4.4E-11	2.9	X	X	X	↑
P01008	Antithrombin-III	3.8E-06	9.7E-06	2.8		X	X	↑
P02647	Apolipoprotein A-I	7.0E-09	3.6E-08	3.8	X	X	X	↑
P02652	Apolipoprotein A-II	1.1E-08	5.0E-08	4.4	X	X	X	↑
P06727	Apolipoprotein A-IV	9.4E-07	2.8E-06	3.0		X	X	↑
P02749	Beta-2-glycoprotein 1	4.0E-05	7.9E-05	1.8		X	X	↑
P07339	Cathepsin D	9.0E-12	7.7E-11	4.6	X	X	X	↑
P00450	Ceruloplasmin	1.9E-06	5.4E-06	2.6	X	X	X	↑
P10909	Clusterin	1.0E-04	1.8E-04	2.0			X	↑
P0C0L4	Complement C4-A	3.0E-05	6.2E-05	3.3				↑
P00751	Complement factor B	1.2E-05	2.7E-05	2.1		X	X	↑
Q9UBP4	Dickkopf-related protein 3	3.5E-04	5.4E-04	1.8				↑
P06396	Gelsolin	5.7E-07	1.8E-06	1.5	X	X		↑
P22352	Glutathione peroxidase 3	1.2E-09	9.1E-09	3.1		X	X	↑
P02790	Hemopexin	7.5E-05	1.4E-04	2.5			X	↑
P01876	Immunoglobulin heavy constant alpha 1	1.7E-04	2.7E-04	3.3		X	X	↑
P01857	Immunoglobulin heavy constant gamma 1	1.1E-04	1.9E-04	2.1			X	↑
P01860	Immunoglobulin heavy constant gamma 3	6.0E-06	1.5E-05	2.7		X		↑
P01834	Immunoglobulin kappa constant	3.5E-13	4.4E-12	6.8	X	X	X	↑
P19827	Inter-alpha-trypsin inhibitor heavy chain H1	2.2E-09	1.4E-08	3.3	X	X	X	↑
Q14624	Inter-alpha-trypsin inhibitor heavy chain H4	6.5E-05	1.2E-04	1.8		X		↑
P01042	Kininogen-1	3.7E-08	1.4E-07	2.7		X	X	↑
P02750	Leucine-rich alpha-2-glycoprotein	2.1E-03	3.1E-03	0.5				↓
Q9UBM4	Opticin	1.9E-05	3.9E-05	2.1		X		↑
P05155	Plasma protease C1 inhibitor	3.6E-09	2.0E-08	2.4	X	X	X	↑
P10745	Retinol-binding protein 3	5.4E-03	7.5E-03	2.5	X	X		↑
P10523	S-arrestin	2.9E-08	1.1E-07	0.1		X	X	↓
P02787	Serotransferrin	2.5E-03	3.6E-03	1.6			X	↑
P02768	Serum albumin	2.0E-06	5.4E-06	2.1		X	X	↑
P02766	Transthyretin	2.6E-07	8.8E-07	2.7		X	X	↑
P02774	Vitamin D-binding protein	7.4E-06	1.7E-05	2.1		X	X	↑
P04004	Vitronectin	2.9E-13	4.4E-12	2.7	X	X	X	↑

The table does not contain regulations, where a protein only is identified in one of the groups (on/off regulations).

X defines proteins in the SRF subgroups with significant differential expression compared to vitreous. The full list of differential regulated proteins in the subgroup analysis can be found in Supplementary Table S4. V, vitreous.

**Figure 3.**
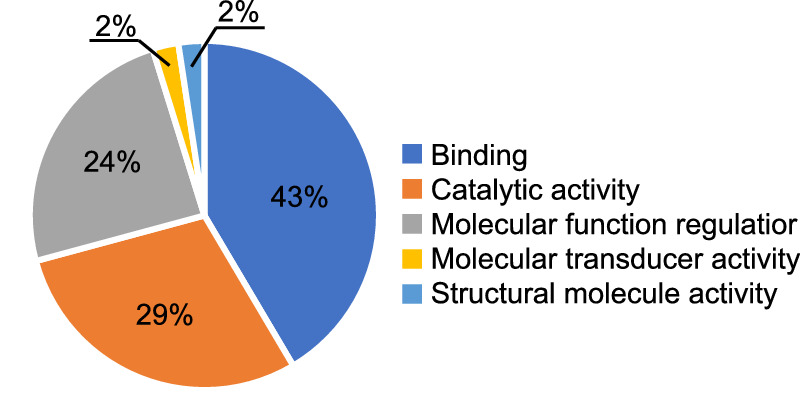
Distribution of the molecular functions of LFQ regulated proteins. The 38 regulated proteins were subjected to functional classification using the PANTHER Classification System. Proteins were grouped into five molecular function categories: binding (GO: 0005488), catalytic activity (GO: 0003824), molecular function regulator (GO: 0098772), molecular transducer activity (GO: 0060089), and structural molecule activity (GO:0005198).

The LFQ regulated proteins (Table 3) were analyzed using the PANTHER Classification System v.15.0.^13^ The statistical overrepresentation test found the blood coagulation pathway to be enriched by 36-fold (*P* = 9.66E-05, FDR = 1.58E-02) in SRF compared to the human background, represented by the three proteins antithrombin III, kininogen-1, and alpha-2-macroglobulin. Furthermore, the molecular functional classification distributed the LFQ regulated proteins into five functional groups: binding (43%), catalytic activity (29%), molecular function regulation (24%), molecular transducer activity (2%), and structural molecule activity (2%) (Fig. 3). Looking at the molecular function using the GO slim annotation revealed a high degree of proteins involved in proteolysis and inhibition hereof (Table 4). Combined, this suggests that proteolysis in relation to the coagulation may be important in the progression of RD.

**Table 4.** GO Slim Annotated Proteins Involved in Proteolysis

Accession	Name	Terminology	LFQ
Proteases			
P00747	Plasminogen	Serine protease	
P00734	Prothrombin	Serine protease	
P00738	Haptoglobin	Serine protease	
P07339	Cathepsin D	Aspartic protease	X
P02790	Hemopexin*	Metalloprotease	X
Protease inhibitors			
P01008	Antithrombin-III	Serine protease inhibitor	X
P01024	Complement C3	Serine protease inhibitor	
P01019	Angiotensinogen	Serine protease inhibitor	X
P01009	Alpha-1-antitrypsin	Serine protease inhibitor	
P05546	Heparin cofactor 2	Serine protease inhibitor	
P05155	Plasma protease C1 inhibitor	Serine protease inhibitor	X
P29622	Kallistatin	Serine protease inhibitor	
P36955	Pigment epithelium-derived factor*	Serine protease inhibitor	
P08185	Corticosteroid-binding globulin*	Serine protease inhibitor	
P01011	Alpha-1-antichymotrypsin	Serine protease inhibitor	X
P05543	Thyroxine-binding globulin	Serine protease inhibitor	
P08697	Alpha-2-antiplasmin	Serine protease inhibitor	
P0C0L4	Complement C4-A	Serine protease inhibitor	X
Inter-alpha-inhibitors		Serine protease inhibitor	
P02760	Protein AMBP		
P19827	Inter-alpha-trypsin inhibitor heavy chain H1		X
P19823	Inter-alpha-trypsin inhibitor heavy chain H2		
Q14624	Inter-alpha-trypsin inhibitor heavy chain H4		X
P04196	Histidine-rich glycoprotein	Protease inhibitor	
P01023	Alpha-2-macroglobulin	Protease inhibitor	X
P02765	Alpha-2-HS-glycoprotein	Protease inhibitor	X
P01042	Kininogen-1	Protease inhibitor	X

SRF proteins identified in the mascot search involved in proteolysis.

Asterisk defines proteins without the canonical enzymatic or inhibitory function of their classes.

LFQ denotes proteins classified as proteases or protease inhibitors in the LFQ analysis (Table 3).

### Mascot Protein Identification

A Mascot search against the SwissProt human database was performed to obtain protein identifications beyond the limit of quantification and using a different search algorithm than used by MaxQuant. Applying no criteria to filter data, that search resulted in 265 protein identifications across SRF samples and 737 protein identifications within the vitreous group. However, to increase confidence in protein identifications, criteria relying on the identification of a protein in a minimum of 50% of the samples in each group, as well as each protein identification represented with a minimum of two peptides, were applied accordingly (see Supplementary Table S2 for all protein identifications with and without the 50% identification [ID] requirement). Protein identifications were then distributed by 147 proteins solely identified in vitreous, 37 proteins unique to SRF, and 61 proteins shared between the two groups. Applying an even more stringent filter of only looking at proteins identified in at least 50% of the SRF samples to all samples of vitreous (no minimum requirement) resulted in identification of 649 proteins in the vitreous humor and 98 proteins in the SRF (Fig. 2). Only five proteins, including chitinase-3-like protein 1 (UniProt: P36222), complement C1q subcomponent subunit C (UniProt: P02747), vasorin (UniProt: Q6EMK4), galectin-3-binding protein (UniProt: Q08380), and cadherin-related family member 1 (UniProt: Q96JP9), were not shared between vitreous and SRF. The five proteins were also uniquely quantified in SRF of the iBAQ analysis, whereas only chitinase-3-like protein 1 and galectin-3-binding protein were uniquely identified across the iBAQ, LFQ, and Mascot analyses (Table 2). Hence, these two proteins may be of particular importance concerning the development of RD.

Subjecting the total SRF Mascot-identified proteins to the overrepresentation test using the PANTHER Classification System, a 34-fold enrichment was found within glycolysis (*P* = 8.88E-10, FDR = 7.28E-08), a 28-fold enrichment (*P* = 3.09E-17, FDR = 5.06E-15) of the blood coagulation pathway, and a 27-fold enrichment (*P* = 3.76E-07, FDR = 1.23E-05) of the plasminogen activating cascade, represented by proteins involved in proteolysis of the two systems. The relevance of proteolysis in the SRF groups was further supported by GO slim molecular function classification of all Mascot-identified SRF proteins highly represented by the two categories: serine-type endopeptidase activity and endopeptidase inhibitor activity (Table 4). Together with the overrepresentation test of the LFQ differentially expressed proteins, this points at proteolysis being an important parameter of the SRF and thus plays crucial role in the RD.

## Discussion

Given the difficulty in obtaining SRF, most proteomic studies have until recently relied on the characterization of vitreous humor in relation to RD. So far, only a few studies have characterized SRF using proteomics.^14^^,^^15^ In the most comparable and recent study, Kowalczuk and colleagues^14^ used a TMT-labeling MS approach comparing two SRF samples from RRD patients with SRF isolated from one patient with central serous chorioretinopathy. They identified a total of 291 proteins, of which 128 were significantly misregulated. Comparing our results of the iBAQ analysis and Mascot ID search to the pilot study by Kowalczuk et al.,^14^ a 76% overlap in protein identifications can be found. The remaining 24% proteins solely identified in our study are all, with few exceptions, top 12 serum proteins removed during the sample preparation in Kowalczuk et al.^14^ Our data involving a larger sample size (24 patients) corroborate the findings in the pilot study of the SRF protein profile. We performed no depletion of highly abundant serum proteins during sample preparation, as we intended to estimate the molar fraction of proteins in the two groups without biasing the data. Furthermore, several of the top 12 serum proteins identified are involved in the regulation of proteolysis, which may play a central role in RRD. While gaining information on the molar fraction of proteins in each group, our approach consequently suffers from proteomic depth, which could be obtained by depleting the most abundant proteins. Thus, future studies using a depletion approach may allow identification of low abundant differentially expressed proteins in SRF compared to vitreous humor.

The protein composition of SRF is difficult to study due to the nature of the RRD. Under normal circumstances, there is no excess liquid between layers of the retina, as seen in detached retina, and thus no apparent control exists. We assume that the initial tear in the neurosensory retina leads to an influx of vitreous humor to the subretinal space and, in that respect, included vitreous from postmortem non-RD eyes as controls. We decided to use postmortem vitreous humor to obtain samples with no history of eye pathology, which would otherwise need to be accounted for, if nonmortem vitreous samples are used. A previous study examining 1- to 7-day postmortem vitreous samples found similar protein profiles throughout the postmortem time intervals,^16^ supporting the use of 2-day postmortem vitreous used within this study. In addition, vitreous is sequestered from blood vessels and contains only few cells, which work in favor of slow protein turnover and little proteolytic contamination from cell lysis.

The protein profile of SRF most likely is dynamic over time due to its encounter with the RPE, thereby provoking the cells to differentiate and migrate as detachment progresses, but also as a consequence of blood–retinal barrier instability. Therefore, the SRF protein profile may be highly reliant on the state of RRD progression. The assessment of protein concentration in the undiluted SRF samples with the vitreous from control eyes revealed a considerable divergence between the two groups. The average concentration in SRF samples was close to 14 times higher than the average vitreous concentration (17.9 vs. 1.2 mg/mL). Additionally, the deviation in protein concentration fluctuated to a much larger extent between SRF samples than in the vitreous samples. The protein concentration of postmortem vitreous used in this study was approximately 2.5 times less than the protein concentration estimated for vitreous isolated from patients with RD.^17^ Assuming that vitreous protein concentration increases in patients with RD, there still exists a considerable gap to the protein concentration of SRF measured in this study. The considerable discrimination in protein concentration within the SRF group may reflect the severity of RRD or the state of progression. However, no apparent correlation was seen in our subgroup analysis dividing the SRF samples into three groups defined by the time interval from RD diagnosis to surgery itself.

Even though our proteomic comparison of SRF to vitreous showed a high resemblance, the molar fraction of total crystallin proteins revealed a large discrimination. The crystallin proteins found as the 50 most abundant proteins in the vitreous included alpha-crystallin A2 chain (UniProt: A0A140G945), alpha-crystallin B chain (UniProt: P02511), beta-crystallin B2 (UniProt: P43320), beta-crystallin S (UniProt: P22914), alpha-enolase (also known as tau-crystallin) (UniProt: P06733), and glyceraldehyde-3-phosphate dehydrogenase (UniProt: P04406). In addition, beta-crystallin B1 (UniProt: P53674), beta-crystallin A4 (UniProt: P53673), Ketimine reductase mu-crystallin (UniProt: Q14894), beta-crystallin B3 (UniProt: P26998), and lambda-crystallin homolog (UniProt: Q9Y2S2) were quantified in the vitreous humor. Together, their molar fraction accounted for 12% of all proteins quantified in vitreous, whereas none of the crystallins were quantified in the SRF. This supports the idea of a selective influx of fluid beneath the neurosensory retina. Disregarding the crystallins in the iBAQ analysis shifts the molar percentage of vitreous protein closer to the values for SRF. We, therefore, suggest that the main protein constituents of the SRF mirror that of the vitreous. The functional classification and overrepresentation test identified proteins involved in the coagulation cascade and proteolysis as important part of the SRF group. These protein groups have previously been shown to be upregulated in another vitreoretinal disease such as proliferative diabetic retinopathy.^18^ Consequently, upregulated SRF proteins belonging to these categories, in our study, may reflect the protein profile of vitreous humor in patients with RD. Furthermore, such proteins may not necessarily be unique to RD but instead be a common feature of vitreoretinal diseases.

The effect of the vitreous humor on the RPE cells has been explored in several studies showing the transition of RPE cells to a mesenchymal phenotype resembling fibroblast-like cells.^19^^,^^20^ Once differentiated, the fibroblast-like cells start migrating and secreting extracellular matrix proteins in fine agreement with the pathology seen in PVR. Our finding that the protein concentration of SRF deviates to a high degree from that of the vitreous may, therefore, be an important determinant of the pace of RPE cell differentiation and migration and thus reflect upon the potential severity state of RD.

Previous studies have shown that particular proteins such as inflammation-associated proteins (alpha-1-antitrypsin, apolipoprotein A-IV, serum albumin, and serotransferrin) become elevated in many vitreoretinal diseases and represent a set of proteins that are probably unique to specific vitreoretinal disease.^15^ In our analyses, two proteins were found only in the SRF samples and in none of the 20 vitreous samples. Galectin-3-binding protein has been previously found secreted by primary cultured RPE cells with elevated levels observed for RPE cells obtained from patients with age-related macular degeneration (AMD).^21^ Although its biological functions are not well defined, it is involved in cell-cell adhesion.^22^ As the name implies, galectin-3-binding protein can interact with galectin 3, and additionally, it can also bind galectin 1. The galectins are involved in cell growth, adhesion, differentiation, inflammation, and apoptosis.^22^ These biological processes may be regulated by galectin-3-binding protein in terms of galectin 1 and 3. Our proteomic data reveal that galectin-1 was quantified in all vitreous samples in the iBAQ analysis (Supplementary Table S2) but none of the SRF samples. Thus, stimulation of RPE cells with galectins (e.g., galectin 1) may be critical in the later stages of RRD, when cells encounter vitreous influx, a process possibly involving galectin-3-binding protein as a regulatory modulator.

The chitinase-3-like protein 1 is a member of the 18 glycosyl hydrolase (GH 18) gene family that has been conserved over species and time and is dysregulated in inflammatory, infectious, remodeling, and neoplastic disorders. The molecule plays a critical role in controlling cell death, inflammation, and remodeling and binds to interleukin (IL) 13 receptor a2 (IL-13Ra2) in a multimeric complex with IL-13Ra2 and IL-13. Furthermore, chitinase-3-like protein 1 can activate macrophage mitogen-activated protein kinase, protein kinase B/AKT, and Wnt/b-catenin signaling and regulate oxidant injury, apoptosis, pyroptosis, inflammasome activation, antibacterial responses, melanoma metastasis, and TGF-b1 production via IL-13Ra2–dependent mechanisms. Together with IL-6, serum concentrations of chitinase-3-like protein have been recently discovered as a novel biomarker for diabetic macular edema with serous RD.^23^^,^^24^

The separation of the neurosensory retina from the RPE layer may be aided by uncontrolled proteolysis. Our data are highly represented by proteases and protease inhibitors, of which several are upregulated in SRF. Most of the proteases (plasminogen and prothrombin) and inhibitors (antithrombin-III, alpha-1-antitrypsin, alpha-1-antichymotrypsin, plasma protease C1, kallistatin, alpha-2-antiplasmin, inter-alpha-inhibitor, and alpha-2-macroglobulin) are classical plasma proteins. However, we find a more than fourfold significant upregulation of cathepsin D in SRF, which makes it the 20th most abundant protein in SRF according to the molar fraction and not observed within the 50 most abundant proteins in the vitreous humor. Cathepsin D has normally been ascribed a ubiquitous role in phagolysosome degradation and has been found highly expressed in the RPE, where it is crucial for proper removal of the continually renewed outer segment.^25^ Dysfunction in the phagolysosomal system has been suggested to be involved in different pathologies of the retina (e.g., AMD),^26^ and cathepsin D–deficient mice were compromised in metabolic maintenance of retinal photoreceptor cells.^27^ Cathepsin D has catalytic activity optima at acidic pH, as found in lysosomes, but also displays activity at neutral pH.^28^ The pH of SRF in our study, however, had a slightly basic pH between 7 and 10 (Supplementary Figure S1), corresponding to pH of the vitreous humor. Thus, the effect of increased cathepsin D in SRF might be insignificant in uncontrolled proteolysis in RD.

In conclusion, the proteome of the SRF is highly represented by proteins involved in proteolysis. Chitinase-3-like protein 1 and galectin-3-binding protein appear to be unique components of the SRF. Such proteins can possibly serve as targets in modulating the effects of SRF in RD.

The mass spectrometry proteomics data have been deposited to the ProteomeXchange Consortium via the PRIDE^29^ partner repository with the data set identifier PXD019159.

## Supplementary Material

Supplement 1

Supplement 2

Supplement 3

Supplement 4

Supplement 5
