# Supplementary material for: Protein Composition of the Subretinal Fluid Suggests Selective Diffusion of Vitreous Proteins in Retinal Detachment
Source: Transl Vis Sci Technol. 2020 Oct 14;9(11):16. doi: 10.1167/tvst.9.11.16 (PMC7571286; doi:10.1167/tvst.9.11.16)
Supplement: Supplement 1 [file tvst-9-11-16_s001.pdf]

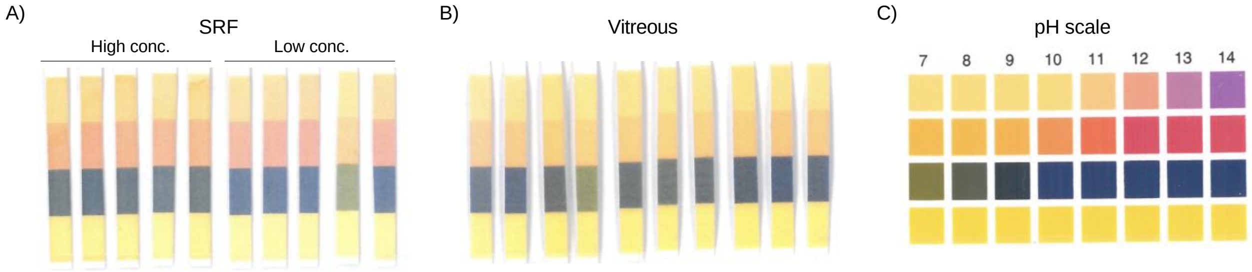

SI Figure 1. pH of SRF and the vitreous humor are basic. A) pH of ten of the SRF samples was measured using pH strips (MColorpHast, Millipore). Measurements included five of the high protein concentration samples and five of the low protein concentration samples. The observed pH range, when compared to pH scale provided by the supplier (C), was pH = 7-10, however, nine of the ten samples were closer to pH 10. B) pH of ten vitreous humor samples was similarly basic in pH. The pH range was 7-9; thus vitreous humor appears to have a slightly narrow pH range than SRF.
