# Supplementary material for: Protein Composition of the Subretinal Fluid Suggests Selective Diffusion of Vitreous Proteins in Retinal Detachment
Source: Transl Vis Sci Technol. 2020 Oct 14;9(11):16. doi: 10.1167/tvst.9.11.16 (PMC7571286; doi:10.1167/tvst.9.11.16)
Supplement: Supplement 2 [file tvst-9-11-16_s002.docx]

**SI Table 1.** Patient data.

| **Patient data for subretinal fluid samples** | | | | |
| --- | --- | --- | --- | --- |
| **Nr. of sample** | **Gender** | **Age** | **Time from RD to surgery*** | **Concentration mg/mL** |
| 1 | Male | 79 | 60 days | 8.0 |
| 2 | Male | 71 | 7 days | 0.9 |
| 3 | Male | 71 | 21 days | 61.0 |
| 4 | Male | 58 | 90 days | 55.8 |
| 5 | Male | 79 | 14 days | 4.5 |
| 6 | Female | 86 | 3 days | 0.5 |
| 7 | Male | 55 | 6 months | 46.5 |
| 8 | Male | 70 | 14 days | 12.1 |
| 9 | Male | 61 | >6 months | 14.2 |
| 10 | Female | 58 | 14 days | 1.0 |
| 11 | Male | 78 | 30 days | 1.1 |
| 12 | Male | 54 | 30 days | 51.8 |
| 13 | Female | 71 | 21 days | 10.1 |
| 14 | Male | 68 | 14 days | 1.5 |
| 15 | Female | 61 | 14 days | 1.0 |
| 16 | Male | 82 | 10 days | 1.5 |
| 17 | Female | 68 | 3 months | 52.5 |
| 18 | Male | 69 | 2 months | 18.3 |
| 19 | Male | 67 | 14 days | 0.0 |
| 20 | Male | 81 | 6 days | 11.3 |
| 21 | Female | 64 | 7 days | 1.0 |
| 22 | Male | 65 | 20 days | 15.8 |
| 23 | Male | 61 | >2 months | 54.8 |
| 24 | Male | 62 | 7 days | 5.2 |

*Since the exact duration of retinal detachment in some cases was not possible to obtain the duration was determined from the moment the patient first noticed significant visual loss or as documented on any available previous ophthalmologic examination report.

| **Patient data for 2-days post-mortem vitreous samples** | | | |
| --- | --- | --- | --- |
| **Nr. of sample** | **Gender** | **Age** | **Concentration mg/mL** |
| 1 | Female | 86 | 1.8 |
| 2 | Male | 90 | 0.7 |
| 3 | Male | 89 | 1.5 |
| 4 | Female | 88 | 0.8 |
| 5 | Female | 82 | 1.7 |
| 6 | Female | 86 | 1.6 |
| 7 | Male | 94 | 1.6 |
| 8 | Male | 73 | 1.2 |
| 9 | Female | 89 | 0.8 |
| 10 | Female | 73 | 0.9 |
| 11 | Male | 79 | 1.4 |
| 12 | Female | 103 | 0.8 |
| 13 | Female | 88 | 1.1 |
| 14 | Male | 95 | 1.4 |
| 15 | Male | 69 | 0.9 |
| 16 | Female | 85 | 1.0 |
| 17 | Male | 76 | 2.0 |
| 18 | Female | 70 | 1.6 |
| 19 | Male | 79 | 1.4 |
| 20 | Female | 70 | 0.6 |
